# Supplementary material for: Content-rich biological network constructed by mining PubMed abstracts
Source: BMC Bioinformatics. 2004 Oct 8;5:147. doi: 10.1186/1471-2105-5-147 (PMC528731; doi:10.1186/1471-2105-5-147)
Supplement: Additional File 2 — The original results of the above study (non-essential files are deleted to keep the file size under the limit set by BMC bioinformatics). [file 1471-2105-5-147-S2.bz2 › chilibotAdditionalFile2/dip10/84ID8139691E300/html/EPHA2_EFNA1.html]

 


 **EPHA2** and **EFNA1** 
  
Found 25 abstracts in PubMed, retrieved 10.  
 

 What does Google say? 
 PDF only 
| .edu only 

---

**Interactive relationship** (e.g. stimulation, inhibition, etc)

**Neutral relationship**- These results suggest that the Eck  [ **EPHA2** ]  B61  [ **EFNA1** ]  interaction, a target of regulatory peptides, plays a role in intestinal epithelial cell development, migration, and barrier function, contributing to homeostasis and preservation of continuity of the epithelial barrier.  Ref: 9357823 Am J Physiol, 1997
- Ephrin A1  [ **EFNA1** ]  ligand was expressed in both tumor and endothelial cells, and  **EphA2**  receptor was localized primarily in tumor associated vascular endothelial cells.  Ref: 12370823 Oncogene, 2002
- Ephrin A1  [ **EFNA1** ]   **EFNA1**  is a GPI anchored ligand that preferentially binds to the receptor tyrosine kinase,  **EphA2** .  Ref: 11741747 Cancer Lett, 2002

**Non-interactive relationship** (e.g. studied together, co-existance, homology, etc.)

- Expression and regulation of Eck  [ **EPHA2** ]  and its cognate ligand B61  [ **EFNA1** ]  were analyzed in the human colonic adenocarcinoma cell line Caco 2.  Ref: 9357823 Am J Physiol, 1997
- EPH A2, or ECK  [ **EPHA2** ]  a receptor for ephrin A1  [ **EFNA1** ] , is ectopically expressed in most melanoma cell lines.  Ref: 10502726 Int J Cancer, 1999
- The expression of other members of this gene family, including ephrin B2,  **EphA2** , and ephrin A1  [ **EFNA1** ] , in a compartmentalized pattern within the Purkinje cell layer suggests a possible redundancy and or a compensation of EphA4 function in the segmental patterning of cerebellar Purkinje cells.  Ref: 11978390 Brain Res Dev Brain Res, 2002
- Cytokines interleukin 1beta IL 1beta, basic fibroblast growth factor, IL 2, epidermal growth factor, and transforming growth factor beta modulated steady state levels of Eck  [ **EPHA2** ]  and B61  [ **EFNA1** ]  mRNA and regulated Eck  [ **EPHA2** ]  activation as assessed by tyrosine phosphorylation.  Ref: 9357823 Am J Physiol, 1997
- These results indicated that autocrine stimulation of  **EphA2**  by  **EFNA1**  could trigger an indirect growth signal by overcoming contact inhibition.  Ref: 11741747 Cancer Lett, 2002
- These data suggest that ephrin A1  [ **EFNA1** ]  and  **EphA2**  play a role in human cancers, at least in part by influencing tumor neovascularization.  Ref: 11146556 Oncogene, 2000
- Functionally, stimulation of Eck  [ **EPHA2** ]  by B61  [ **EFNA1** ]  resulted in increased proliferation, enhanced barrier function, and enhanced restitution of injured epithelial monolayers.  Ref: 9357823 Am J Physiol, 1997
- Epithelial cell kinase  [ **EPHA2** ]  B61  [ **EFNA1** ]  an autocrine loop modulating intestinal epithelial migration and barrier function.  Ref: 9357823 Am J Physiol, 1997
- **EphA2**  and ephrin A1  [ **EFNA1** ]  are members of the Eph family of receptor tyrosine kinases and ligands, which are implicated in a number of developmental processes.  Ref: 11641774 Oncogene, 2001
- Following addition of  **EFNA1**  Fc to HT29 cells, tyrosine hyperphosphorylation of  **EphA2** , E cadherin, and beta catenin were observed.  Ref: 11741747 Cancer Lett, 2002
- Strong tumor cell expression of FLT 4, ephrin A1  [ **EFNA1** ] , and  **EphA2**  was associated with increased melanoma thickness, and ephrin A1  [ **EFNA1** ]  staining was related to decreased survival P = 0.039.  Ref: 11891198 Am J Pathol, 2002
- Whether activation of  **EphA2**  by  **EFNA1**  is involved in aberrant growth or differentiation of cancer cells is currently not known.  Ref: 11741747 Cancer Lett, 2002
- By immunohistochemistry, the ephrin A1  [ **EFNA1** ]  ligand and one of its receptors,  **EphA2** , were detected throughout tumor vasculature.  Ref: 11146556 Oncogene, 2000
- Importance of vascular phenotype by basic fibroblast growth factor, and influence of the angiogenic factors basic fibroblast growth factor fibroblast growth factor receptor 1 and ephrin A1  [ **EFNA1** ]   **EphA2**  on melanoma progression.  Ref: 11891198 Am J Pathol, 2002
- Ephrin A1  [ **EFNA1** ]  and  **EphA2**  were also detected in both the vasculature and tumor cells of surgically removed human cancers.  Ref: 11146556 Oncogene, 2000
- Within one minute after stimulation of  **EphA2**  with its ligand, ephrin A1  [ **EFNA1** ] , the protein tyrosine phosphatase SHP2 is recruited to  **EphA2** .  Ref: 10655584 Nat Cell Biol, 2000
- Immunocytochemical staining demonstrated coexpression of Eck  [ **EPHA2** ]  and B61  [ **EFNA1** ]  in the same cells, suggestive of an autocrine loop.  Ref: 9357823 Am J Physiol, 1997
- The ephrin A1  [ **EFNA1** ]  ligand and its receptor,  **EphA2** , are expressed during tumor neovascularization.  Ref: 11146556 Oncogene, 2000
- Interestingly, the ligand for  **EphA2** , ephrin A1  [ **EFNA1** ] , is also regulated by p53.  Ref: 11641774 Oncogene, 2001
- ... the ephrin A1  [ **EFNA1** ]   **EphA2**  pathway might also be important for tumor cell proliferation and patient survival.  Ref: 11891198 Am J Pathol, 2002
- Furthermore,  **EphA2**  was tyrosine phosphorylated in the xenograft tumors, indicating that it was activated, presumably by interacting with ephrin A1  [ **EFNA1** ] .  Ref: 11146556 Oncogene, 2000
- Double labeling with anti CD34 antibodies demonstrated that both ephrin A1  [ **EFNA1** ]  and  **EphA2**  were expressed in xenograft endothelial cells and also tumor cells.  Ref: 11146556 Oncogene, 2000
- Remarkably, the oncogenic properties of  **EphA2**  contravene traditional dogma with regard to the oncogenic properties of a growth factor and its receptor tyrosine kinase while stimulation of  **EphA2**  by its ligand ephrin A1  [ **EFNA1** ]  results in  **EphA2**  autophosphorylation, the stimulation reverses the oncogenic transformation.  Ref: 12242697 Microsc Res Tech, 2002
